# Analyzing and Modelling the Corrosion Behavior of Ni/Al_2_O_3_, Ni/SiC, Ni/ZrO_2_ and Ni/Graphene Nanocomposite Coatings

**DOI:** 10.3390/ma10111225

**Published:** 2017-10-25

**Authors:** Mian Hammad Nazir, Zulfiqar Ahmad Khan, Adil Saeed, Vasilios Bakolas, Wolfgang Braun, Rizwan Bajwa, Saqib Rafique

**Affiliations:** 1 NanoCorr Energy and Modelling Research Group (NCEM), Bournemouth University Talbot Campus, Poole, Dorset BH12 5BB, UK; hnazir@bournemouth.ac.uk (M.H.N.); rbajwa@bournemoth.ac.uk (R.B.); 2 Global College of Engineering and Technology, CPO Ruwi 112, Muscat Sultanate P.O. Box 2546, Oman; asaeed@gcet.edu.om; 3 Advanced Bearing Analysis, Schaeffler Technologies AG & Co. KG, 91074 Herzogenaurach, Germany; vasilios.bakolas@schaeffler.com (V.B.); Wolfgang.Braun@schaeffler.com (W.B.); 4 Low Dimensional Materials Research Centre, University of Malaya, Kuala Lumpur 50603, Malaysia; saqibrafique@hotmail.com

**Keywords:** nanocomposite coatings, nanoparticles, corrosion, analysis, modelling, simulation

## Abstract

A study has been presented on the effects of intrinsic mechanical parameters, such as surface stress, surface elastic modulus, surface porosity, permeability and grain size on the corrosion failure of nanocomposite coatings. A set of mechano-electrochemical equations was developed by combining the popular Butler–Volmer and Duhem expressions to analyze the direct influence of mechanical parameters on the electrochemical reactions in nanocomposite coatings. Nanocomposite coatings of Ni with Al_2_O_3_, SiC, ZrO_2_ and Graphene nanoparticles were studied as examples. The predictions showed that the corrosion rate of the nanocoatings increased with increasing grain size due to increase in surface stress, surface porosity and permeability of nanocoatings. A detailed experimental study was performed in which the nanocomposite coatings were subjected to an accelerated corrosion testing. The experimental results helped to develop and validate the equations by qualitative comparison between the experimental and predicted results showing good agreement between the two.

## 1. Introduction

This paper enables researchers to fully understand the complex relationship between the mechanical properties and macroscopic phenomena, including electrochemical and chemical responses of nanocoatings. It is well known that nanomaterial has large surface-to-volume ratio compared to bulk material, however, in the case of nanocoating this role becomes even more significant [1]. A literature survey shows that the classical electrochemical theory does not fully incorporate the influences of mechanical parameters of nanocoatings [2,3].

It has been found in the literature [4,5] that the combined effects of both the mechanical and electrochemical parameters directly influence the electrochemical reactions on the surface of nanocoating. Khan-Nazir [6,7] suggested that electrochemical reactions on metal coating surfaces are stress-dependent. Sometimes, both mechanical and electrochemical effects, coupled together, result in the modified corrosion rates of the nanocoating compared to its stress-free state [8,9,10]. Therefore, it can be established that the size-dependent electrochemical properties of nanocoatings would lead to size-dependent surface stress [11,12]. Studies suggest that for nanograined coatings, the electrochemical reaction rate is greatly size-dependent. For example the corrosion resistance properties of Ni (Nickel) in salt solution were significantly improved with the reduction in grain size to the nanometer scale [13,14,15].

Although, both Ni sputtered nanocrystalline and Ni bulk coatings have the same chemical composition, the breakdown voltage of sputtered coating is higher than that of the bulk Ni coating [16,17]. The reason for higher corrosion resistance of the sputtered coating is its smaller grain size. Mishra [18] also reported that the electrochemical and corrosion behavior of nanocrystalline Ni of different grain sizes (8–28 nm) had a higher breakdown potential due to finer grain size than that of coarse-grained polycrystalline bulk Ni. Freshly exposed nanocrystalline Ni offered higher corrosion resistance due to larger hindrance to anodic dissolution compared to that of bulk Ni. Contrarily, the corrosion resistance of the Cu_90_Ni_10_ alloy was reduced with the reduction in grain size to the nanometer scale [19,20]. Therefore, it is essential to understand the size-dependent electrochemical properties of nanocoatings, which are the central points in the current study. The nanocoatings which have been analysed in this work are nanocomposite structures [21]. This means that they are composed of two or more immiscible components, of which at least one is restricted to the nanometer scale such as composites of nanoparticles Al_2_O_3_ with Ni forming Ni/Al_2_O_3_ composite coating. A nanocomposite coating is formed by a matrix where filler nanoparticles are dispersed.

Corrosion reaction rates are normally modelled by utilizing the Butler–Volmer expression [22,23]. The conventional Butler–Volmer expression does not include the influence of surface stress on corrosion reactions. Khan-Nazir [24,25], analyzed the influence of the applied stress on the electrochemical reaction of bulk material coating in a step-wise manner. In the present study, the Gibbs–Duhem expression [26,27] for calculating the stress-dependent chemical potential was utilized to modify the conventional Butler–Volmer expression. By combining the Gibbs–Duhem [26,28] and Butler–Volmer [4] expressions in the form of combined mechano-electrochemical equations, the reliance of the electrochemical reaction on the intrinsic surface elastic parameters, such as surface stress, surface elastic modulus, surface porosity, permeability and average maximum grain size was obtained. The intrinsic surface mechanical properties were combined to simulate the size-dependent electrochemical properties, for instance the corrosion rate of coatings.

This paper discusses a detailed experimental study which was conducted by using four different types of nanocomposite coatings: Ni/Al_2_O_3_, Ni/SiC, Ni/ZrO_2_ and Ni/Graphene (GPL). These coatings were subjected to accelerated corrosion testing to analyze the effects of distinct mechanical properties of each the coatings on their corrosion behaviors. These effects were analyzed and compared by using surface characterization methods, including scanning electron microscopy (SEM), energy-dispersive X-ray spectroscopy (EDS), X-ray diffraction (XRD), and corrosion rate monitoring methods including electrochemical impedance spectroscopy (EIS). Based on the observations recorded from experimentation, a set of combined mechano–electrochemical equations were developed to predict the corrosion rates of nanocomposite coatings. Finally, the equations were validated by qualitative comparison between experimental and predicted results.

## 2. Experiment

### 2.1. Sample Preparation

All composite coatings Ni/Al_2_O_3_, Ni/SiC, Ni/ZrO_2_ and Ni/Graphene (GPL) were pulse electrodeposited with a thickness of h = 5 µm over circular steel disc substrates. The diameter of all circular discs was 40 mm with naked substrate roughness of 0.05 μm. Prior to each coating deposition, the substrate surface conditioning was performed using deionized water and acetone under ultrasonic treatment.

The Watt’s-type bath solutions were prepared for developing different types of composite coatings. The solution compositions consisted of NiSO_4_6H_2_O (265 g/L), NiCl_2_6H_2_O (48 g/L) and H_3_BO_3_ (31 g/L). Four types of nanoparticles with an amount of 20 g/L were ultrasonically dispersed in different solutions. The sizes of nano-particles were: Nano Al_2_O_3_ (50–60 nm), Nano SiC (40–50 nm), Nano ZrO_2_ (30–40 nm) and Graphene platelets (6–8 nm). The solutions had been magnetically stirred overnight to yield better particle suspension prior to the start of the deposition process. The pH of the solution was adjusted to between 4.0 and 4.5 by sodium hydroxide or diluted sulphuric acid and recorded using a Tecpel pH meter.

The electrodeposition process parameters were kept constant as current density (3 A/dm^2^) and pulse on–off time (20–80 ms) with a duty cycle of 20%. A high-quality nickel sheet was used as an anode, and a steel circular disc of 80 mm diameter and 8.20 mm thick was used as a cathode.

### 2.2. Accelerated Corrosion Testing

All the coated samples were exposed to salt spray testing for 450 h. Salt spray testing was performed by placing samples in a test chamber which was operated according to the specified conditions in ASTM B117. This test was performed to analyze the relative resistance to corrosion of coated samples when exposed to a salt spray climate at elevated temperature. The chamber was enclosed with a well-maintained temperature of +35 °C, and a continuous indirect spray of 5% aqueous sodium chloride solution was allowed on to the samples at a specified rate.

The appearance of corrosion products was evaluated after the exposure test. Surface characterization of test samples, including crystal orientation and grain size, was performed both pre-exposure and post-exposure using SEM (JEOL USA Inc., Peabody, MA, USA), EDS (JEOL USA Inc., Peabody, MA, USA) and XRD (BRUKER, ,Petaling Jaya, Malaysia).

### 2.3. Experimental Observations

#### 2.3.1. Surface Corrosion and Porosity

**Figure 1:**
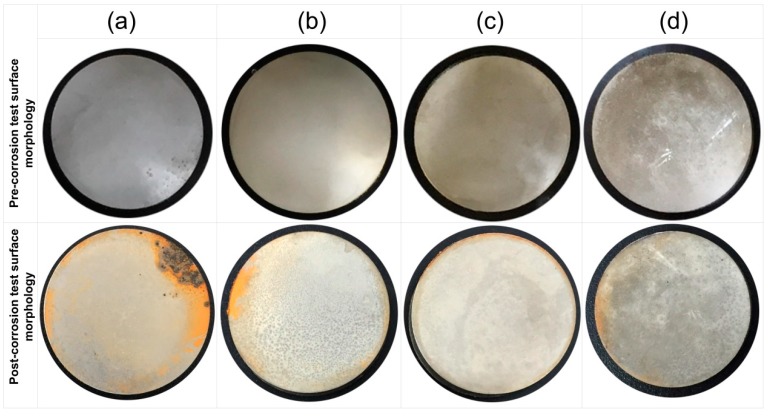
Corrosion status of electrodeposited composite coatings immersed in 5% NaCl solution after 450 h; (**a**) Ni/Al_2_O_3_ (**b**) Ni/SiC (**c**) Ni/ZrO_2_ and (**d**) Ni/GPL.

Figure 1 shows the corrosion status of electrodeposited composite coatings after 450 h of salt spray exposure. The images clearly show that the highest surface corrosion was found for the case of Ni/Al_2_O_3_ coating which subsequently decreased for the case of Ni/SiC and Ni/ZrO_2_ coatings. The lowest surface corrosion was found for the case of Ni/GPL coating. According to the latest studies [29], the first five atomic surface layers of nano coatings have the highest degradation rates because of direct exposure to the environment. Therefore, prominent changes in the surface morphology of post-test samples can be seen from the images.

In Table 1, the pre-exposure SEM micrographs for all the composite coatings demonstrate compacted fine-deposit grain microstructure while the pre-exposure EDS micrographs for the coatings identify the elements and their concentrations. The SEM results showed that Ni/Al_2_O_3_ nano-sized particles revealed bigger deposit grain size when compared to the remainder coatings. This also contributed to an increase in the average surface roughness of Ni/Al_2_O_3_ coating with a value of 0.21 μm followed by Ni/SiC (0.19 μm), Ni/ZrO_2_ (0.06 μm) and Ni/GPL (0.007 μm) coatings respectively. The reason for large surface roughness behavior can be attributed to the existence of bigger particles on the surface. With the decrease in the particle size from Ni/Al_2_O_3_ to Ni/GPL, the surface roughness of coating decreased. Also, large particle size in Ni/Al_2_O_3_ coatings accounted for large porous structure. The decreasing particle size from Ni/Al_2_O_3_ to Ni/GPL coating resulted in the formation of fine coating with minimum porosity. For example, Ni/GPL coating which had the smallest particle size formed a very fine glass-like coating with minimum porosity.

The post exposure SEM and EDS results revealed highest corrosion products (Fe = 3.0%) appearance in Ni/Al_2_O_3_ and smallest corrosion product (Fe = 0.3%) appearance in Ni/GPL coating. This behavior can be directly linked to the porosity level which sets the Oxygen and NaCl electrolyte permeability of coatings. Post exposure micrographs showed that Oxygen and electrolyte permeability of coatings decreased with a decrease in the particle size. The inhibition efficiency was found to increase with decreasing the size of particles showing that Ni/GPL coating had the least corrosion found after exposure.

#### 2.3.2. Crystallinity and Grain Size

**Figure 2:**
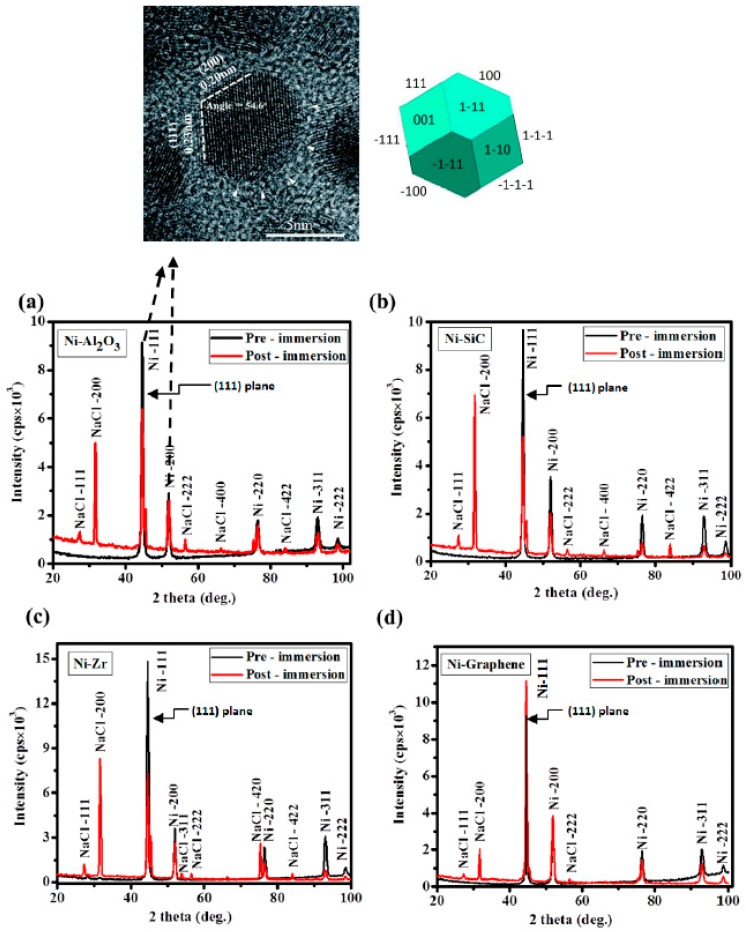
The XRD patterns of Ni coatings doped with (**a**) Al_2_O_3_; (**b**) SiC; (**c**) ZrO_2_ and (**d**) GPL.

Figure 2 shows the XRD patterns of all the composite coatings before and after exposure to the salt spray testing. The pre exposure XRD patterns of all coatings (black peaks) show cubic crystal structure with ‘strong’ diffraction peaks at 44.51° and 51.85°, corresponding to the reflection from (111) and (200) plane, respectively, as shown in the top microscopic image Figure 2. Whereas, relatively low intensity peaks at 76.37°, 92.95° and 98.45° are due to the reflection from the (220), (311) and (222) planes, respectively. On the other hand, the post exposure results (red peaks) show the new peaks corresponding to the NaCl crystal structures emerged in the XRD patterns for all variants of coatings. These peaks at ~27.34°, 31.7°, 56.41° and 75.30° correspond to the cubic symmetry of NaCl crystal structure and ascribed to the (111), (200), (222), (420) and (422) crystal planes, respectively. There were no significant changes recorded in any of the spectra, however, intensity of the peaks (especially (111) and (200) peaks) after the immersion reduced for all the samples in order to compensate the externally diffused NaCl crystal peaks. This behavior is attributed to the increase in porosity and reduction in the inhibition efficiency of coatings. For the case of Ni/GPL, minimum drop in the intensity peaks in addition to the few peaks for NaCl was observed compared to the other coatings.

**Figure 3:**
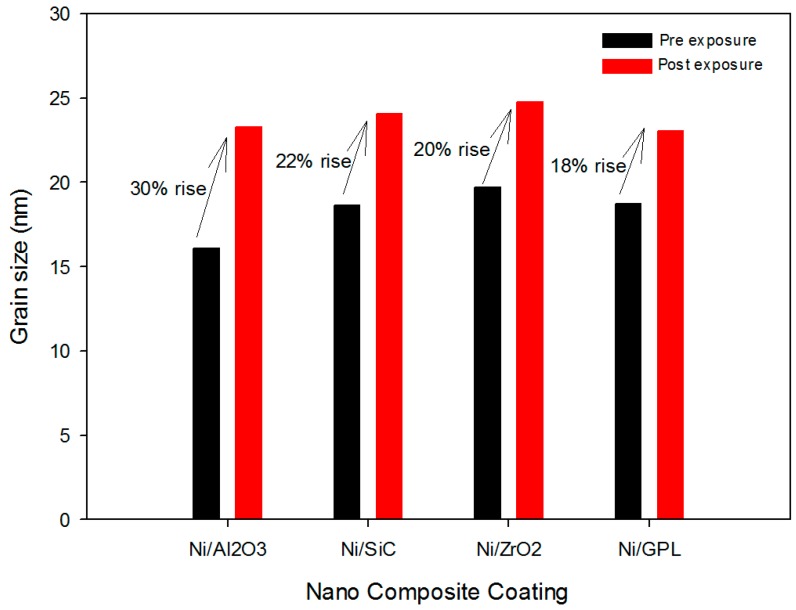
Grain size as a function of different nano composite coatings.

Further investigation showed that the highest % rise in the grain size after exposure was found in the case of Ni/Al_2_O_3_ coating, while the lowest % rise was observed in case of Ni/GPL coating, as shown in Figure 3. The large % rise in Ni/Al_2_O_3_ occurred due to its open, disordered nature of grain boundaries, meaning that NaCl crystals diffused more rapidly down boundaries leading to more rapid coble creep [30]. Since grain boundaries are regions of high energy, they make excellent sites for the nucleation of salt precipitates. Contrarily, Ni/GPL coating had compact, refined grain structure leading to least diffusion of NaCl crystals.

#### 2.3.3. Surface Stress and Corrosion Rate

**Figure 4:**
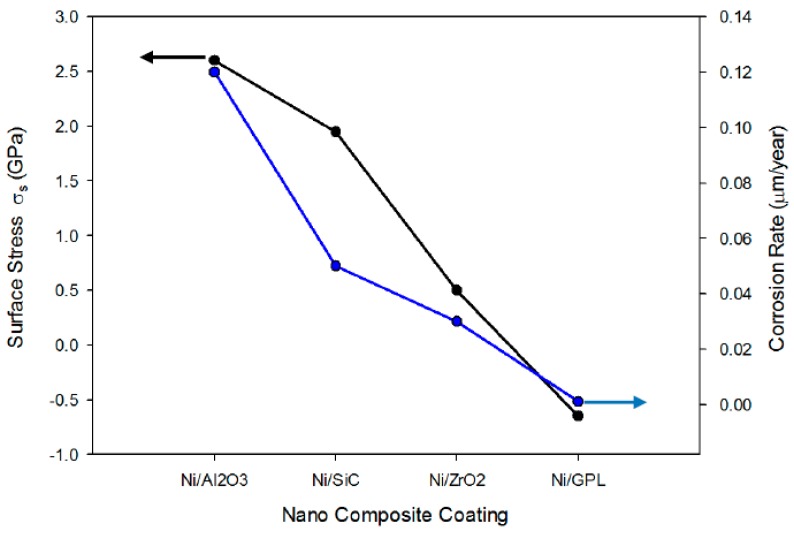
Surface Stresses and Corrosion rate of different nanocomposite coatings.

The residual stresses in all the coatings were measured by XRD and Reuss model using the computer program, Stress [16,31,32], while the corrosion rate measurement was performed by EIS measurements using potentiostat setup. From Figure 4 it was established that the nano particle size affects both the residual stresses and the corrosion rate of nickel-based composite coatings. The larger particles of Ni/Al_2_O_3_ impeded the lateral growth of the nickel layer, producing a weak (111) plane compared to the rest of the coatings, which resulted in high tensile residual stresses and large porosity. Large tensile stress with high porosity led to the large openings of micro pores in Ni/Al_2_O_3_, providing easy pathways for NaCl ions to diffuse through the pores. This behavior, commonly known as ‘diffusion creep’ resulted in a high intergranular corrosion rate of Ni/Al_2_O_3_. Similar tensile behavior was also found in the case of Ni/SiC and Ni/ZrO_2_, but rather in a decreasing order. On the other hand, the finer particles of Ni/GPL resulted in compressive stresses and minimum porosity, ultimately leading to the minimum corrosion rate.

## 3. Mathematical Model

**Figure 5:**
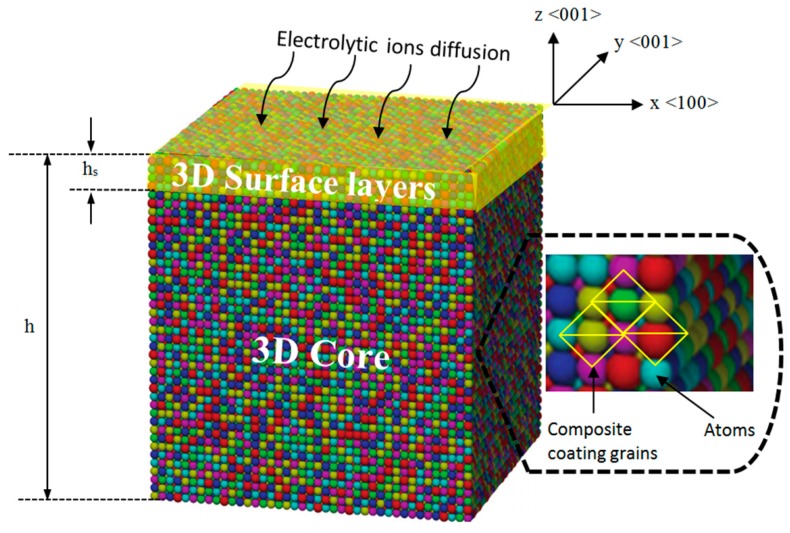
A 3D model of nanocomposite coating separated into a 3D surface layer with the thickness h_s_ coherently adhered to the 3D core with the thickness h − h_s_.

A set of mechano-electrochemical equations was developed for the nanocomposite coating. The nanocomposite coating model consists of the 3D surface layers adhered to a 3D core (Figure 5). It is assumed that the surface layers and the core are linearly elastic and mechanically isotropic in addition to homogenous stresses. The <100>, <010> and <001> lattice directions represent the *x*, *y* and *z* coordinates respectively. This model develops, in a step-wise manner, the thermodynamic equations for:

- Mechanical parameters including surface stress, porosity, permeability, particle size and grain size of the composite coating.
- Corrosion rate of the composite coating.

The focus of study is on the elastic properties of a composite coating with typical three-dimensional (3D) structure to simplify the theoretical analysis. In the study of surface thermodynamic properties of composite materials, the 3D surface layer is assumed to be 1 nm thick. This assumption follows the fact that during the relaxation process of nanocomposite coating, only the first five atomic surface layers (shown as yellow in Figure 5) encounter normal relaxation, while the rest of the atomic layers remain undeformed [29,33]. After this, the surface thickness is assumed to be 1 nm to model the size-dependent properties of the coating.

### 3.1. Mechanical Parameters Modelling

Usually, 3D surfaces are modelled using the diffusive and interphase surface approaches [34]. The diffusive interface represents the concentration gradient of electrolyte [35]. The interface is treated as a thermodynamic phase by the interphase approach with no significant variation of properties along the interface. As the atoms within the 1 nm thin surface layer face a very distinct local environment (e.g., corrosive) compared to the core, the mechanical response and physical properties of the surface will be different from those of the core.

Consider a composite coating which has debonded from the stress-free substrate; the coating will reach its minimum energy after relaxing and attaining equilibrium. There are two types of relaxation processes, including normal and parallel relaxation [9,36]. The normal relaxation leads to the creation of an eigenstress $\sigma_{s}^{o}$ in the surface layer, while the normal relaxation leads to creation of a stress (or initial strain $\varepsilon_{\mathrm{ini}}$) in the core to balance the surface stress. As the surface is bonded to the core, both the surface and core should undergo the same magnitude of deformation.

If the coating state after normal relaxation is taken as the reference configuration, the total potential energy of a coating is given by [37]:

(1) $$U\left(\varepsilon \right)=2h_{s}L_{o}^{2}u_{s}\left(\varepsilon \right)+\left(h-2h_{s}\right)L_{o}^{2}u_{c}\left(\varepsilon \right)$$

where $h_{s}$ is the surface layer thickness and h is the overall thicknesses, ε is the strain (bi-axial), $L_{o}$ is the length and width of the coating, $u_{s}$ is the energy density of the surface layer and $u_{c}$ is the energy density of the core. Both $u_{s}$ and $u_{c}$ can be written as [37]:

(2) $$u_{s}\left(\varepsilon \right)=u_{s}^{o}+2\;\sigma_{s}^{o}\;\varepsilon +Y_{s}\varepsilon^{2}$$

(3) $$u_{c\left(\varepsilon \right)}=u_{c}^{o}+Y_{c}\varepsilon^{2}$$

where $u_{s}^{o}$ is the strain-free energy density of the surface layer, $u_{c}^{o}$ is strain-free energy density of the core, $Y_{c}$ is the bulk biaxial Young’s modulus; $Y_{s}$ is the 3D surface biaxial Young’s modulus and $\sigma_{s}^{o}$ is the 3D surface eigenstress.

Substituting Equations (2) and (3) into Equation (1) give the potential energy of a coating as:

(4) $$U\left(\varepsilon \right)=2h_{s}L_{o}^{2}\left(u_{s}^{o}+2\sigma_{s}^{o}\;\varepsilon +Y_{s}\varepsilon^{2}\right)+\left(h-2h_{s}\right)L_{o}^{2}\;\left(u_{c}^{o}+Y_{c}\varepsilon^{2}\right)$$

At the equilibrium, the energy minimization requires $\partial {\frac{U\left(\varepsilon \right)}{\partial \varepsilon }|}_{\varepsilon =\varepsilon^{\mathrm{ini}}}=0$, which yields the generalized Young-Laplace Equation [38] to describe the mechanical force balance between the surface layer and the core:

(5) $$2h_{s}\sigma_{s}^{o}+2h_{s}Y_{s}\varepsilon^{\mathrm{ini}}+\left(h-2h_{s}\right)Y_{c}\varepsilon^{\mathrm{ini}}=2F_{s}^{\mathrm{ini}}+F_{c}^{\mathrm{ini}}=0$$

where $F_{c}^{\mathrm{ini}}=\left(h-2h_{s}\right)Y_{c}\varepsilon_{\mathrm{ini}}$ is the force on core and $F_{s}^{\mathrm{ini}}=h_{s}\left(\sigma_{s}^{o}+Y_{s}\varepsilon_{\mathrm{ini}}\right)$ is the force on surface.

The solution of Equation (5) gives the initial strain which is induced by the surface eigenstress as,

(6) $$\varepsilon_{\mathrm{ini}}=\frac{2h_{s}\sigma_{s}^{o}}{2h_{s}Y_{s}+\left(h-2h_{s}\right)Y_{c}}$$

Equation (6) can be used to calculate the biaxial surface stress $\sigma_{s}$ of the coating by using Hooke’s law as,

(7) $$\sigma_{s}=\sigma_{s}^{o}+Y_{s}\varepsilon^{\mathrm{ini}}=\left[1-\frac{2h_{s}Y_{s}}{2h_{s}Y_{s}+\left(h-2h_{s}\right)Y_{c}}\right]$$

It should be noted that the biaxial surface stress of the coating depend on the thickness h of the coating. Surface stress and Young’s modulus can be used to calculate the surface porosity $P_{s}$ of coating as [39].

(8) $$P_{s}=\gamma_{s}\exp\left(-\frac{\sigma_{s}}{Y_{s}}\right)$$

where $\gamma_{s}$ is the stress sensitivity coefficient [40].

Surface porosity of coating can be related to the surface permeability $k_{s}$ of coating as [41].

(9) $$k_{s}=\beta P_{s}^{m}$$

where β and m are material constants for coating.

Permeability and Porosity can be used to calculate the nano particle size (diameter) $D_{p}$ using modified form of Darcy’s law as [42]

(10) $$D_{p}=\sqrt{\frac{{\left(1-P_{s}\right)}^{2}\;k_{s}}{P_{s}^{3}}}$$

The relation between average maximum grain size *D*_max_ with the particle radius $\left(\frac{D_{p}}{2}\right)$ and its volume fraction (*f*) in the nano composite coatings is given as [43];

(11) $$D_{\max}=D_{o}+\frac{D_{p}}{2}\sqrt{\frac{8}{f}}$$

Average maximum grain size is the average size of grains after deformation has occurred, while the deposit grain size $D_{o}$ is the size of grain at the time of deposition. It is noteworthy that Equation (11) was derived by assuming that particles would not be separated from grain boundaries even when the moving grain boundaries (due to external pressure) drag particles during the relaxation stage.

### 3.2. Corrosion Rate Modelling

The electrochemical reaction of the surface of coating is written as:

(12) $$M↔M^{Z+}+Ze^{-}$$

where $M^{Z+}$ and M are the metal oxidised and metal reduced forms and Z represents the number of transferred electrons. From the anodic sites on the coating surface the ions are dissolved in the electrolyte and the current flows between the sites and the electrolyte.

For coating in a homogenous electrolyte solution, the current simultaneously faces the influence of two external factors: mechanical and electrical. Considering this, it is possible to write the electrochemical potential of the corresponding particles by using the modified form of Gibbs–Duhem expression as [4,26]:

(13) $${\overset{↔}{µ}}_{C}={µ}_{\Delta P=0}+ZF\Phi +\Delta PV_{m}={µ}_{o}+RT\ln a+ZF\Phi +\Delta PV_{m}$$

where ${µ}_{\Delta P=0}$ is the chemical potential for zero pressure, $V_{m}$ is the coating molar volume, T is the temperature, Φ is the electrical potential, a is the activity coefficient, ${µ}_{o}$ represents the standard potential (when a = 1) and F is the Faraday constant [44].

In equilibrium, for Equation (12), the Gibbs free energy ΔG = 0, therefore using Equation (13) ΔG can be written as,

(14) $$\Delta G={\bar{µ}}_{C}^{Z+}+{Z\bar{µ}}_{e^{-}}-{\bar{µ}}_{C}={µ}_{C}^{Z+}+{Zµ}_{e^{-}}-{µ}_{C}-\mathrm{ZF}\left(\Phi_{C}-\Phi_{\mathrm{sol}}\right)-{\Delta \mathrm{PV}}_{m}=0$$

where $\Phi_{C}$ is the electrical potential of the coating, $\Phi_{\mathrm{sol}}$ is the electrolyte potential. From Equation (14), the equilibrium potential of the surface reaction corresponding to pressure ΔP is given as [4,45,46]:

(15) $${\overset{↔}{\Phi }}_{e}=\Phi_{M}-\Phi_{\mathrm{sol}}=\frac{{µ}_{M}^{Z+}+{Zµ}_{e}-{µ}_{M}-{\Delta \mathrm{PV}}_{m}}{\mathrm{ZF}}=\Phi_{e}-\frac{{\Delta \mathrm{PV}}_{m}}{\mathrm{ZF}}$$

When any mechanical deformation occurs on the coating, the coating surface will undergo a change in chemical/mechano-electrochemical potential, as per Equation (13), denoted as ${\Delta \mathrm{PV}}_{m}$.

The current density due to the surface electrochemical reactions accompanying surface stress can be written by using well known Butler–Volmer expression as [4,47]:

(16) $$\overset{↔}{J}={\overset{↔}{j}}_{f}-{\overset{↔}{j}}_{r}=j_{o}\left[\exp\left(\frac{\alpha ZF\eta +\Delta PV_{m}}{RT}\right)-\exp\left(\frac{-\left(1-\alpha \right)ZF\eta }{RT}\right)\right]$$

where,

(17) $${\overset{↔}{j}}_{f}=j_{o}\exp\left(\frac{\alpha ZF\eta +\Delta PV_{m}}{\mathrm{RT}}\right)$$

(18) $${\overset{↔}{j}}_{r}=j_{o}\exp\left(\frac{-\left(1-\alpha \right)ZF\eta }{\mathrm{RT}}\right)$$

where $j_{o}$ is the equilibrium exchange current density corresponding to equilibrium potential ${\overset{↔}{\Phi }}_{e}$ (η = 0) and α is the charge transfer coefficient. The chemical potential of coating can be changed by applying both tensile and compression stresses, also referred to as hydrostatic stress tensor ΔP. Surface stress is the main factor for the electrochemical reaction rate on the coating surface. By utilizing the biaxial surface stress (σ_s_) of the coating in Equation (7), it is possible to obtain the pressure in terms of stress tensor as ΔP = 2σ_s_/3.

By substituting ΔP into Equations (16)–(18), the current density of the coating can be written as,

(19) $$\overset{↔}{J}={\overset{↔}{j}}_{f}-{\overset{↔}{j}}_{r}=j_{o}\left[\exp\left(\frac{\alpha ZF\eta }{\mathrm{RT}}\right)\exp\left(\frac{2\sigma_{s}^{o}V_{m}}{3\mathrm{RT}}\left[1-\frac{2h_{s}Y_{s}}{2h_{s}Y_{s}+\left(h-2h_{s}\right)Y_{c}}\right]\right)-\left(\exp\left(\frac{-\left(1-\alpha \right)ZF\eta }{\mathrm{RT}}\right)\right)\right]$$

The corrosion rate of metal electrode depends on the current density of the electrochemical reaction of the coating. According to Faraday’s law, there is a linear relationship between the corrosion rate, and the corrosion current density $\overset{↔}{J}$.

(20) $$\mathrm{Corrosion}\;\mathrm{Rate}=K_{1}\frac{\overset{↔}{J}}{\rho }\mathrm{EW}$$

where corrosion rate is given in mm/year, $K_{1}$ is the constant given as 3.27 × 10^−3^ (mm·g)/(µA·cm·year), ρ is the density in g/cm^3^ and EW is the equivalent weight in grams.

## 4. Modelling Results and Discussion

In order to evaluate the intrinsic surface elastic properties of nanocomposite coatings, molecular dynamics (MD) simulations were performed [48]. MD simulations of Ni/Al_2_O_3_, Ni/SiC, Ni/ZrO_2_ and Ni/GPL composite coatings face-centered-cubic crystals utilized the LAMMPS code [49] along with the embedded-atom technique [50]. Furthermore, molecular statics (MS) configuration using the conjugate gradient procedure was used during simulations [51].

A stress-free model of nanocomposite coating with atoms positioned within a lattice was created. The simulations of parameters applying boundary conditions were performed only along x and y directions with free surfaces in the z direction. Unreformed volume, length and width L_0_ and coating thickness h were evaluated using MD simulations [48]. The unreformed volume of coating multiplied by crystal density was used to evaluate the weight of atoms. The range of coating thickness varied between 100 nm to 5 µm along with 8 × 8 unit cells both along x and y coordinates. The coating relaxation was performed in dual step, i.e., at first normal relaxation along z direction and then parallel relaxation along all three directions, in order to accomplish the minimal energy requirement [48,52]. After parallel relaxation and achieving the equilibrium, the energy reaches a minimum level with length of coating as L_ini_. Subsequently, the coating’s initial strain ε_ini_ was calculated by using $\varepsilon_{\mathrm{ini}}=ln\left(L_{ini}/L_{0}\right)$. MD simulations were performed for attaining the 3D intrinsic surface elastic parameters of 3D surface eigenstress $\sigma_{s}^{o}$, 3D surface biaxial Young’s modulus $Y_{s}$ and 3D core biaxial Young’s modulus $Y_{c}$ respectively. These parameters are shown in Table 2, subject to assumption that the surface layer thickness $h_{s}$ = 1 nm.

**Figure 6:**
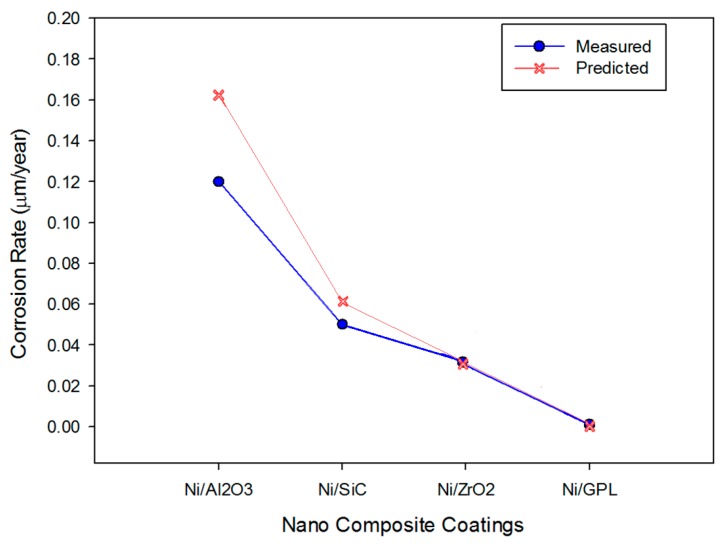
The predicted and measured corrosion rates.

Substituting the values in Table 2 into the developed mechano-electrochemical equations, the corrosion rate for all the coatings can be simulated, as shown in Figure 6. The predicted and measured corrosion rates from experiments were compared to validate the model reliability for all the coatings, as shown in Figure 6. The predicted results of corrosion in NaCl environments are in agreement with the experimental results. In the simulation, the model slightly over-predicts the corrosion rates for some data points, which makes some points in this graph deviate from the measured data.

**Figure 7:**
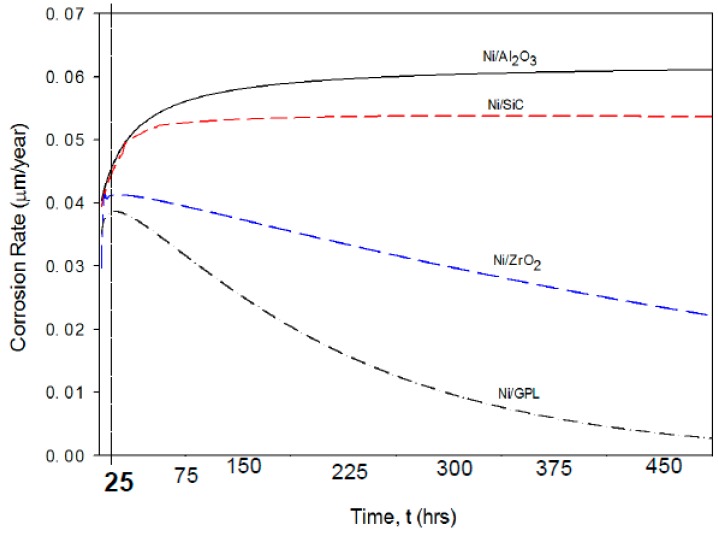
The predicted corrosion rates of nanocomposite coatings with respect to exposure time.

The predicted corrosion rates of Ni/Al_2_O_3_, Ni/SiC, Ni/ZrO_2_ and Ni/GPL with respect to exposure time t are shown in Figure 7. It is clear that the identical corrosion rate curvatures can be observed for all the coatings corresponding to the very short exposure time (i.e., t <25 h). After then, the corrosion rate curvatures reach a maximum and then reduce slowly with passing exposure time, especially in the case of Ni/ZrO_2_ and Ni/GPL. The reason for this reduction in corrosion rate after 25 h is linked to the increasing grain size with passing time. Lowest reduction in corrosion rate was found in the case of Ni/Al_2_O_3_ because the % rise in the grain size for Ni/Al_2_O_3_ was largest (also evident from Figure 3). However, the largest reduction in corrosion rate was found in the case of Ni/GPL because the % rise in the grain size for Ni/GPL was smallest. The larger grain size in Ni/Al_2_O_3_ resulted in open and disordered nature of grain boundaries, which caused much rapid diffusion of NaCl crystals down boundaries, leading to higher corrosion rate. While Ni/GPL had compact and refined grain structure causing least diffusion of NaCl crystals leading to lowest corrosion rate.

**Figure 8:**
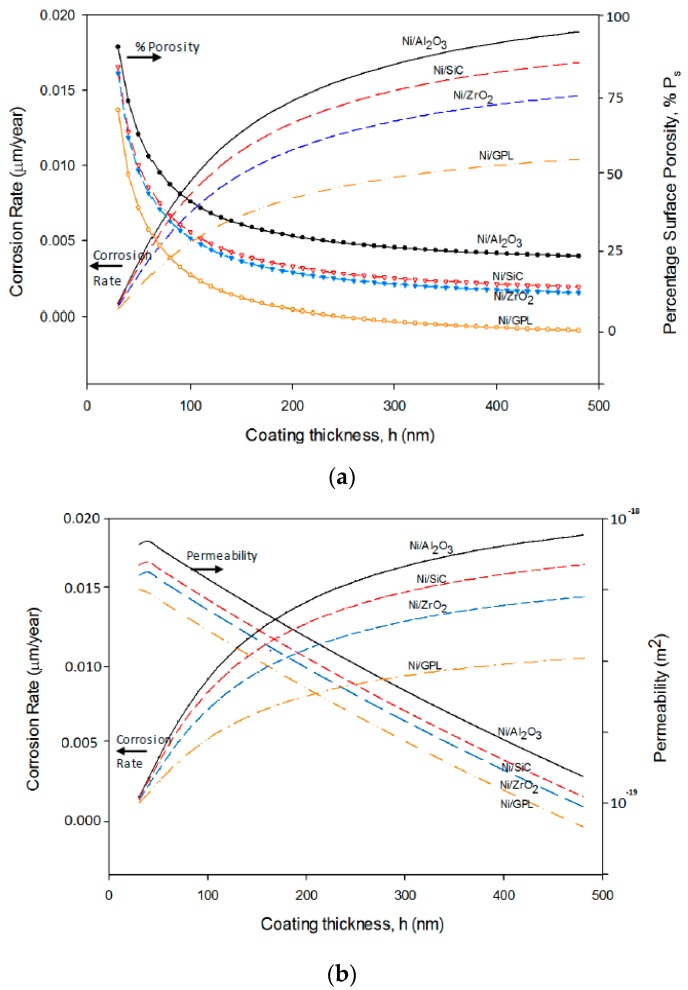
The predicted corrosion rates with respect to coating thickness as a function of (**a**) surface porosity and (**b**) permeability.

The predicted corrosion rates and percentage surface porosity as a function of coating thickness for Ni/Al_2_O_3_, Ni/SiC, Ni/ZrO_2_ and Ni/GPL are shown in Figure 8a. It can be seen from Figure 7 that the porosity of extremely thin coatings (less than about 100 nm) is very high as it is directly affected by substrate texture [53,54]. While at greater thicknesses the slope of the porosity-thickness curve is controlled by parameters relevant to the deposit itself. Between these two regimes is a sharp, well-marked transition region in which the porosity of the deposit falls extremely rapidly. The porosity at any given thickness is affected by the deposit grain size. As Ni/Al_2_O_3_ has the largest deposit grain size, its porosity-thickness curve accounts for highest porosity and a corresponding highest corrosion rate. While Ni/GPL has the smallest deposit grain size its porosity-thickness curve accounts for lowest porosity and a corresponding lowest corrosion rate.

Figure 8b shows the increasing corrosion rate corresponding to decreasing permeability as a function of coating thickness for Ni/Al_2_O_3_, Ni/SiC, Ni/ZrO_2_ and Ni/GPL. This behavior can be directly linked to the porosity level which sets the Oxygen and NaCl electrolyte permeability of coating. As the porosity of coating decreases with the increasing coating thickness, the permeability of Oxygen and electrolyte also decreases with the increasing thickness. Like porosity, the permeability also directly depends on the deposit grain size. Therefore, Ni/Al_2_O_3_, which has largest deposit grain size accounts for the highest permeability and corrosion rate, while Ni/GPL, which has the smallest deposit grain size accounts for the lowest permeability and corrosion rate.

**Figure 9:**
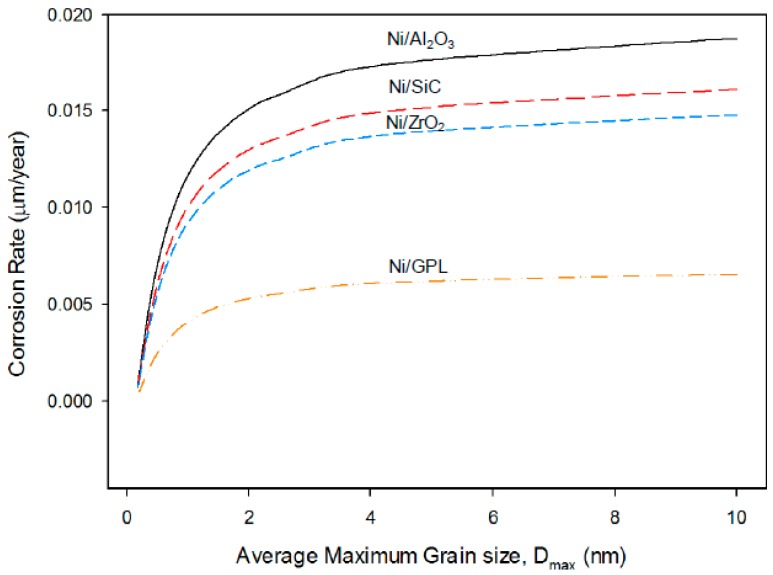
The corrosion rates of coatings as a function of average maximum grain size.

The predictions in Figure 9 show that, for all the coatings, the corrosion rate increases with increasing average maximum grain size where the average maximum grain size is calculated based on the assumption of spherical grains. The increasing average maximum grain size results in the formation of open, disordered nature of grain boundaries. The grain boundaries make excellent sites for the nucleation of salt precipitates leading to intergranular corrosion. As intergranular corrosion is caused by the precipitation of salt in grain boundaries, the corrosion rate is affected by the volume fraction of precipitated salt per unit grain boundary area. It can be seen that for a given average maximum grain size, the highest corrosion rate was observed in the case of Ni/Al_2_O_3_, while the lowest was observed in the case of Ni/GPL. The reason for the associated corrosion rates is that Ni/Al_2_O_3_ accounts for the largest deposit grain size while Ni/GPL accounts for the smallest deposit grain size at the time of deposition.

**Figure 10:**
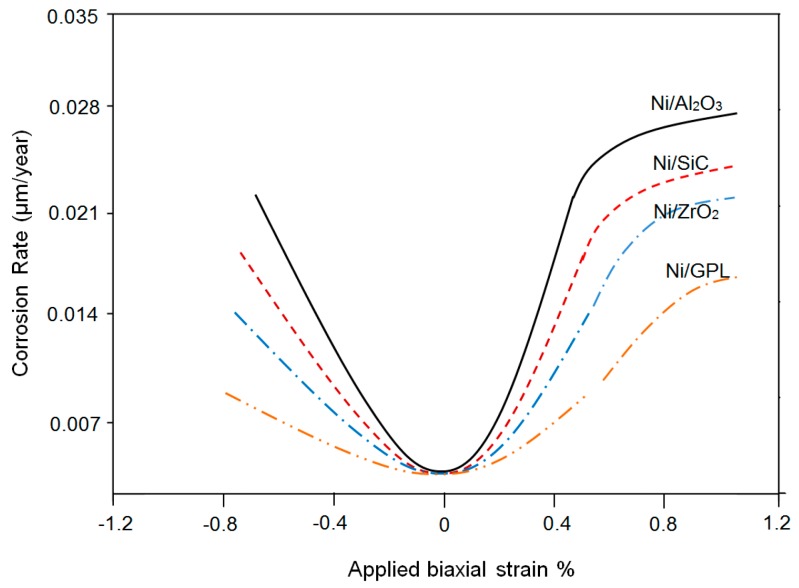
The corrosion rates of coatings as a function of applied biaxial strain.

Figure 10 shows the relation between predicted corrosion rates as a function of applied biaxial strain. During the deformation of coating under the applied compressive strain, the corrosion rate showed a decreasing trend. While contrarily under the applied tensile strain the corrosion rate showed an increasing trend. The corrosion rate decreases under compressive deformation as pores contract, therefore hindering salt diffusion towards intergranular boundaries [44,55]. On the other hand, the corrosion rate increases under tensile deformation as more pores open up, therefore permitting salts to easily diffuse [56]. Ni/Al_2_O_3_ had the highest corrosion rate during compressive and tensile deformations. It is noteworthy that at zero biaxial strain the corrosion rates become minimal, because with no-deformation in the coating system the pores return to normal under stress-free conditions.

## 5. Conclusions

A series of combined mechano-electrochemical equations for predicting the corrosion rate of nanocomposite coatings have been developed, which combines the mechanical properties and electrochemical responses of the nanocomposite coatings. The equations take into account the most important parameters in the corrosion of nanocomposite coatings: surface stresses, grain size, porosity, permeability, equilibrium potential and the anodic current density. An extended form of the popular Butler–Volmer expression, combined with the Gibbs–Duhem expression, was used for analyzing the dependence of the corrosion rate on intrinsic mechanical parameters, such as surface stress, surface elastic modulus, surface porosity, permeability and grain size. The predictions showed that the corrosion rate of the Ni/Al_2_O_3_, Ni/SiC, Ni/ZrO_2_ and Ni/GPL nanocomposite coatings increased with increasing deposit grain size due to increase in surface stress, porosity and permeability. A model experiment, using accelerated corrosion testing, was performed in conjunction with the analysis. The experiment showed good qualitative agreement with the trends predicted by the theory.

## Figures and Tables

**Table 1:** Pre- and Post-exposure surface morphology results.

Method	Pre Exposure	Post Exposure
SEM	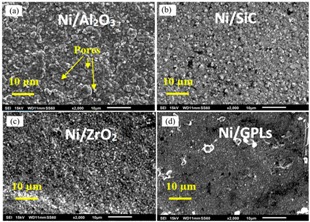	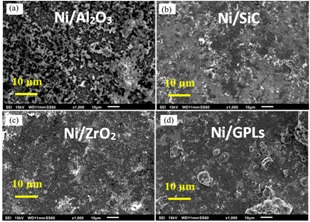
EDS	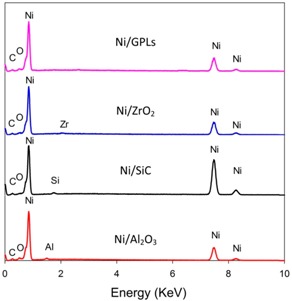	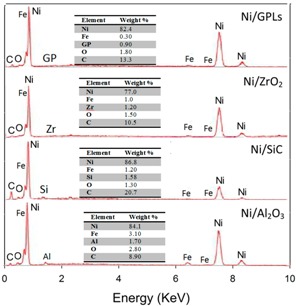

**Table 2:** Fitted parameter values from the MD simulations.

Materials	$\sigma_{s}^{o}$ (GPa)	$Y_{s}$ (GPa)	$Y_{c}$ (GPa)	α	β	m	*D_o_* (nm)
Ni/Al_2_O_3_	2.92	223.12	209.32	0.21	1.5	0.3	35
Ni/SiC	2.15	251.32	240.68	0.17	0.7	1.0	21
Ni/ZrO_2_	2.01	264.22	243.81	0.165	1.3	0.6	18
Ni/GPL	1.98	278.32	252.02	0.13	0.4	1.7	2
